# Not all wounds are visible: Peer bullying and psychopathology in child and adolescent psychotherapy outpatients

**DOI:** 10.1186/s13034-026-01146-w

**Published:** 2026-07-29

**Authors:** Anna-Luisa Kranhold, Sina Weber, Carolin Köhmstedt, Karen Krause, Dieter Wolke, Silvia Schneider, Babett Voigt

**Affiliations:** 1 Mental Health Research and Treatment Centre (FBZ), Clinical Child and Adolescent Psychology, Faculty of Psychology, Ruhr-University Bochum, Massenbergstraße 9-13, 44787 Bochum, Germany; 2 German Centre for Mental Health (DZPG), partner site Bochum/Marburg, Bochum, Germany; 3 Department of Psychology, University of Warwick, Coventry, UK

**Keywords:** Bullying, Mental health, Cognitive behavioural therapy, Child and adolescent psychotherapy

## Abstract

**Background:**

Bullying poses a major risk for poor mental health. While findings from the general population highlight harmful effects of bullying, data from clinical samples with diverse diagnoses and evidence-based diagnostic methods is rare. This study aimed at exploring the association between psychopathology and active bullying involvement in child and adolescent psychotherapy outpatients.

**Methods:**

The preregistered study (https://aspredicted.org/VYW_9TT) included data from *N* = 719 outpatients aged 6–20 years from a German child and adolescent psychotherapy centre. Current experiences as a victim, bully-victim, or bully and distressing bullying-specific intrusions were assessed with a screening instrument. Gold-standard diagnostic assessment of psychopathology provided categorial ICD-10 diagnoses. The Strength and Difficulties Questionnaire depicted a dimensional proxy measure of psychopathology. Analyses included examining the frequencies of the bullying roles (victim, bully-victim, bully) and the occurrence of distressing bullying-specific intrusions, Pearson’s chi-square tests for gender-specific differences, binary logistic regression analyses testing the bullying roles as predictors of ICD-10 diagnoses, and multiple one-way ANOVAs examining group differences on the Strength and Difficulties Questionnaire scores between the bullying roles.

**Results:**

29% of all outpatients reported to be actively involved in bullying (22% victims, 5% bully-victims, 2% bullies), with 32% frequently experiencing distressing bullying-specific intrusions. While victims were more likely to have mood disorders (ICD-10 F3 disorders), bully-victims were less likely to have internalising and more likely to have externalising disorders and behavioural and emotional disorders with onset usually occurring in childhood and adolescence (ICD-10 F9 disorders). Bullies were more likely to have multiple disorders but less likely to have internalising disorders. Independent from specific diagnoses, victims, bully-victims, and bullies overall reported greater emotional and behavioural difficulties than outpatients without bullying experiences.

**Conclusions:**

Among outpatients in child and adolescent psychotherapy, bullying is linked to specific related psychological burden and role-specific psychopathology. Bullying and related psychological burden should be addressed and routinely assessed in mental health care settings. The link between bullying and psychopathology as well as specific intervention methods may be explored further in future research.

**Supplementary Information:**

The online version contains supplementary material available at 10.1186/s13034-026-01146-w.

## Background

The Lancet Psychiatry Commission on youth mental health calls the rising incidence of mental health problems in young people a global crisis and the “continuing neglect” of their needs “intolerable” [1]. The onset of most psychological disorders occurs before adulthood [2], highlighting childhood and adolescence as critical developmental periods for prevention and early intervention in mental health care.

Peer interactions strongly influence the development and well-being of children and especially adolescents [3]. Bullying is a frequent type of negative peer interaction characterised by a behavioural pattern of violence, harmful intention and power imbalance between those involved [4]. Recent large-scale studies report a bullying prevalence of 20% among 15-year-old students internationally (21% in Germany [5]) and 14% involvement in school bullying in 11-15-year-old students in Germany [6]. Bullying is considered a major risk factor for poor mental health and affected children and adolescents frequently rely on health care resources [7–8]. Bullying is not specifically listed as one of the stressors included in the A criterion for post-traumatic stress disorder, therefore, it does not meet the current definition of trauma. However, the debate on how this A criterion should be interpreted and if bullying constitutes one such stressor is still ongoing, as bullying has been identified as a significant risk factor for posttraumatic stress symptoms, such as intrusions [9–10].

A substantial body of research based on general populations demonstrates that active involvement in bullying is associated with increased risk for psychopathology [11–16]. There is a high risk of internalising disorders for former victims and of externalising disorders for former bullies; former bully-victims (i.e., those who experienced both victimisation and perpetration and who were oftentimes not distinguished from bullies in previous studies) are at risk for both internalising and externalising disorders in adulthood [17]. These findings suggest differential associations between bullying roles (victims/bully-victims/bullies) and mental health outcomes, arguing against the conceptualisation of bullying as an entirely unspecific risk factor. As most studies rely on school-based samples, it remains unclear whether these patterns also apply to treatment-seeking populations.

To date, clinical research on bullying in clinical samples remains comparatively scarce. Bullying involvement has been found to be more frequent among children and adolescents receiving psychotherapy than in school-based samples [18–21]. Clinical studies focusing on specific disorders have reported associations between victimisation and anxiety [22], depression [23], posttraumatic stress disorder symptoms [24], borderline personality disorder symptoms [25], internalising problems and suicide attempts [20], and non-suicidal self-injury [26]. These findings support the notion that bullying victimisation may represent a specific risk factor for disorders within the internalising spectrum. However, prior clinical studies have typically focused on specific disorders, neglecting potential associations with other disorders and the differentiation between pure victims, bully-victims, and pure bullies.

Initial findings from the limited number of clinical studies examining all three roles and multiple disorders found that victims have an increased risk for mood disorders [18], overall internalising disorders, chronic somatic conditions, and suicide attempts [27–28], and externalising disorders [21]. Bully-victims showed an elevated risk for externalising disorders [28] and for comorbid internalising and externalising disorders [21]. Bullies were more likely to report low well-being [18], externalising disorders, and suicide attempts [21, 27, 28].

Overall, these findings point toward a rather specific profile among bullies, which aligns with results from school-based studies. In contrast, patterns observed among victims and bully-victims appear more heterogeneous, suggesting that victimisation may constitute a largely unspecific risk factor associated with multiple forms of psychopathology. This partially contradicts findings from school samples and from clinical studies with a narrow diagnostic focus. The reasons for these inconsistencies remain unclear. One major methodological difference between school-based and clinical studies concerns the operationalisation of psychopathology outcomes. Whereas school studies typically rely on dimensional assessments of psychological symptoms or indirect indicators, clinical studies predominantly employ categorical diagnoses. This distinction may substantially influence conclusions regarding the specificity of bullying as a risk factor.

Combining dimensional and categorical measures of psychopathology within a clinical sample may enhance comparability with school samples and provide insight into whether bullying involvement has diagnosis-specific or -unspecific transdiagnostic associations with mental health problems. Investigating bullying and diagnosis-specific and -unspecific outcomes within clinical samples allows for the examination of not only its relations to mental health problems in general, but also its specific link to certain diagnoses. Such an approach may contribute to a more differentiated understanding of bullying and may facilitate bullying-informed assessment and treatment planning in child and adolescent psychotherapy.

### The present study

This study examined a large, unselected sample of outpatients from a German routine child and adolescent outpatient psychotherapy centre. Psychopathology was assessed in terms of specific categorial diagnoses (structured clinical interviews) and a dimensional measure for diagnosis-unspecific emotional and behavioural difficulties (well-established screening), from the children’s and from the caregiver perspective (multi-informant), to inform the following questions:

1. How many child and adolescent psychotherapy outpatients report bullying and distressing bullying-specific intrusions? In addition to the prevalence of bullying victimisation and perpetration, distressing bullying-specific intrusions will be analysed exploratorily as an example of bullying-related psychological burden.
2. Do outpatients with and without experiences of bullying differ in their reported psychopathology?
  - Regarding specific categorial diagnoses, we hypothesised higher likelihoods of internalising diagnoses in victims and higher likelihoods of externalising diagnoses in bullies, victims, and bully-victims compared to outpatients without such bullying experiences, consistent with previous findings [18, 21, 28].
  - Regarding diagnosis-unspecific dimensional emotional and behavioural difficulties, we hypothesised higher levels in bullies, victims, and bully-victims and less prosocial behaviour in bullies and bully-victims compared to outpatients without such bullying experiences, in accordance with previous findings [18, 21, 28].

## Methods

### Participants

The sample comprised *N* = 719 outpatients receiving Cognitive Behavioural Therapy (CBT) in a German child and adolescent outpatient psychotherapy centre. In the German healthcare system, such psychotherapeutic outpatient clinics are part of the routine clinical care system and classified as specialised outpatient mental health care. All patients may access psychotherapeutic consultations without a referral or be referred by other services such as general practitioners, paediatricians, or child and adolescent psychiatrists. Treatments are provided by licensed psychotherapists (or psychotherapists in training under supervision) and covered by statutory health insurance. Medication treatment is not provided within office-based psychotherapeutic practices or outpatient psychotherapeutic clinics but is typically delivered by child and adolescent psychiatrists in separate outpatient or inpatient services. Consistent with other routine CBT services in Germany, the child and adolescent outpatient psychotherapy centre included in this study offers CBT to children and adolescents across the broad range all mental health disorders, provided that outpatient psychotherapy is clinically indicated.

**Fig. 1:**
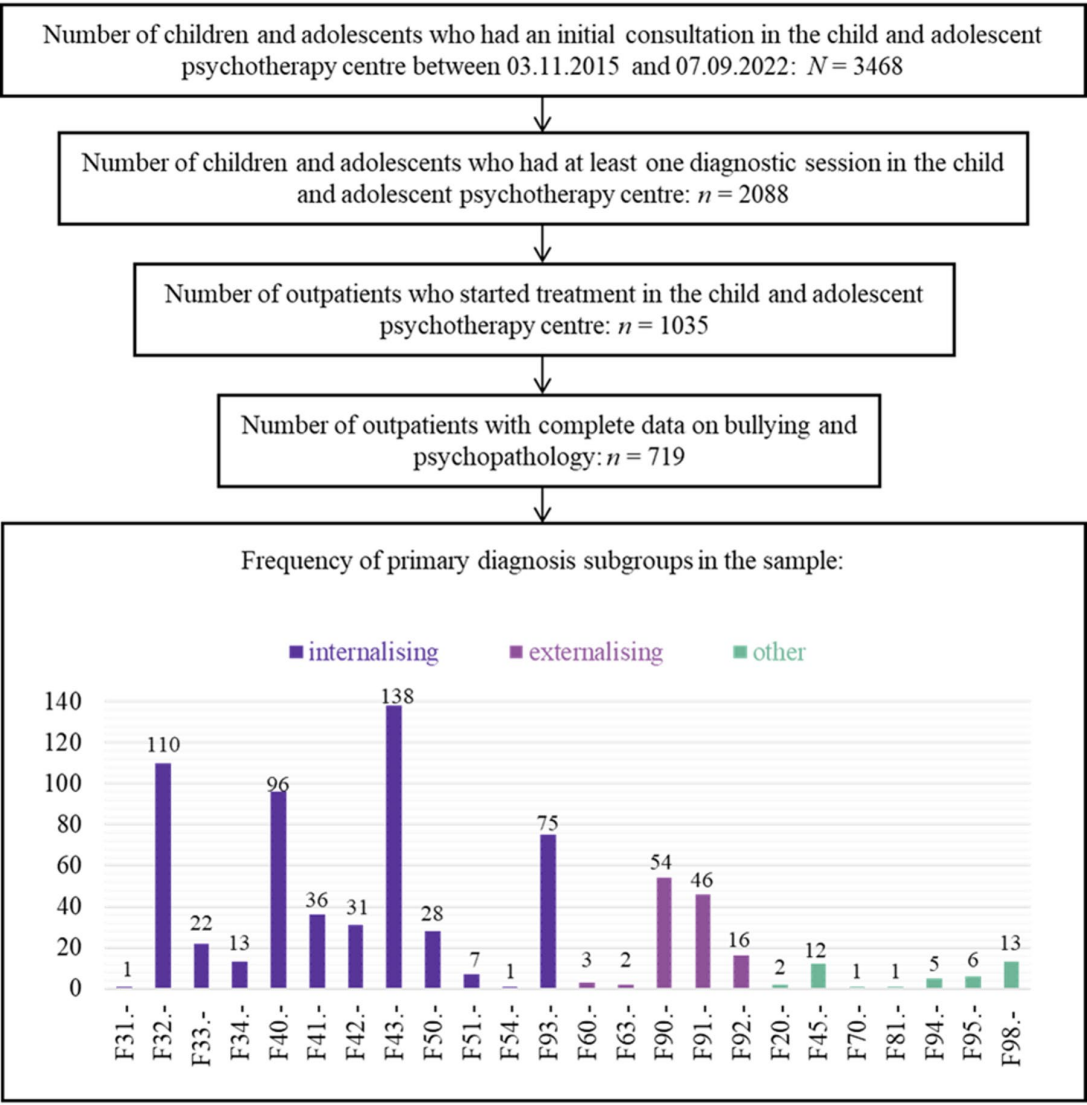
Flow Diagram of the Participant Flow and List of the Primary Diagnoses in the Outpatient Sample Note. ICD-10 diagnoses after supervised structured clinical interviews (self- and caregiver-report) by trained mental health professionals. Primary diagnosis subgroups = categorised as internalising (F31, F32, F33, F34, F40, F41, F42, F43, F50, F51, F54, F93), externalising (F60, F63, F90, F91, F92), and other (F20, F45, F70, F81, F94, F95, F98) according to [34]

Data from a sub-sample (2015–2018) was presented in a previous publication [19]. All outpatients aged 6–20 years were included if they had completed their probationary therapy phase between November 2015 and September 2022, received a diagnosis based on structured clinical interviews and completed questionnaires on bullying and emotional and behavioural problems. The mean age was *M* = 13.31 years (*SD* = 3.28, range = 6–20 years). 58% identified as female and 42% as male. *N* = 114 outpatients were children (ages 6–9) and *n* = 605 were adolescents (ages 10–20). The cross-sectional study was preregistered at AsPredicted (https://aspredicted.org/VYW_9TT) and approved by the Ethics Committee of the Faculty for Psychology at Ruhr-University Bochum (votum 431). Written informed parental consent, adult patients’ written informed consent, and underage patients’ oral informed consent were obtained prior to data collection. Figure 1 shows participant flow and primary diagnoses.

### Instruments

#### The screening for bullying experiences

Peer bullying was assessed with an adaptation of a questionnaire originally developed by Wolke et al. [29, 30]. First psychometric properties of this version can be found in a previous publication [19]. Items included various forms and roles of bullying (direct, indirect, cyber victimisation and perpetration) and reported distressing bullying-specific intrusions (i.e., “Do you regularly have vivid memories of these bullying experiences at school/at home/at university or at work? Do you sometimes relive these experiences and does this cause you distress?”). Patients indicated how often the described issue had occurred during the last six months on the following four-point Likert scale: 0 = never, 1 = rarely (1–3 times), 2 = frequently (more than 4 times), 3 = very frequently (at least once a week). Bullying and distressing bullying-specific intrusions were classified as present for scores greater than 1, lower scores indicated the absence of bullying. Patients were assigned to one of the following roles: victim (victimisation, no perpetration), bully-victim (both victimisation and perpetration), and bully (perpetration, no victimisation). Bullying involvement referred to all patients with experiences as a bully, victim or bully-victim. Direct bullying covered overt behaviour (e.g., physical violence, verbal abuse), indirect bullying involved covert behaviour (e.g., social exclusion, spread rumors), while cyber bullying included digital technologies.

### Diagnostic interview for mental disorders in children and adolescents (Kinder-DIPS)

Categorical diagnoses were obtained using a structured clinical interview for the most common psychological disorders in children and adolescents, the Kinder-DIPS (original title: “Diagnostisches Interview bei psychischen Störungen im Kindes- und Jugendalter“) [31]. Reliability, validity, and acceptance findings have been reported previously [32]. The interviews were conducted by trained and certified independent clinicians with patients and caregivers separately and followed by supervision. The final diagnoses according to ICD-10 [33] were based on the supervised child and caregiver interview results.

Based on the hierarchical taxonomy of psychopathology (HiTOP) [34], the ICD-10 diagnoses were grouped into the following categories: primary diagnosis of F2-F9, internalising primary diagnosis (F31/F32/F33/F34/F40/F41/F42/F43/F50/F51/F54/F93), externalising primary diagnosis (F60/F63/F90/F91/F92), one diagnosis, multiple diagnoses (comorbidity), whereas F20/F45/F70/F81/F94/F95/F98 could not be assigned to internalising or externalising [34].

### The strength and difficulties questionnaire (SDQ)

The SDQ [35] assessed emotional and behavioural difficulties as a dimensional proxy measure of psychopathology. Self- and caregiver-report include five subscales: emotional symptoms, peer problems, hyperactivity/inattention, conduct problems, and prosocial behaviour. All 25 items are rated on a three-point Likert scale (0 = not true, 1 = somewhat true, 2 = certainly true). Scores were calculated for each subscale, the total difficulties, and two frequently applied higher-order factors (externalising/internalising problems). Previous research provides information on the psychometric properties of the German version of the SDQ [36].

### Statistical analyses

Analyses were conducted using R [37]. Preliminary analyses assessed the extent of psychopathology within the sample (ICD-10 diagnoses and SDQ scores). For question 1, relative frequencies of the bullying roles and distressing bullying-specific intrusions were calculated, along with gender-specific differences (Pearson’s chi-square tests). For question 2a, each bullying role (victim/bully-victim/bully vs. not experienced as the reference category) was tested as dichotomous, single predictor of each diagnosis group (dichotomous outcome) separately in binary logistic regression analyses. Outcomes included F2-F9 as primary diagnosis, internalising primary diagnosis, externalising primary diagnosis, one diagnosis, and multiple diagnoses. For question 2b, group differences on the SDQ scores (continuous outcome) were examined for the same three bullying roles in multiple one-way ANOVAs. Outcomes included total difficulties, internalising problems, externalising problems, and all subscales. To control for the increased risk of Type I error due to multiple testing, p-values were adjusted using the Benjamini-Hochberg procedure [38].

## Results

### Preliminary analyses

78% of the sample received internalising primary ICD-10 diagnoses. The most prevalent disorders were: F43 Reaction to severe stress, and adjustment disorders (*n* = 138), F32 Depressive episodes (*n* = 110) and F40 Phobic anxiety disorders (*n* = 96, see Fig. 1 for all frequencies). Table 1 presents frequencies of ICD-10 diagnosis groups within the sample, descriptives for the SDQ can be found in Table 2.

**Table 1:** Frequencies of primary ICD-10 diagnosis groups overall and according to gender

ICD-10 diagnoses	Total (*N* = 719)		Gender			
			Male (*n* = 303)		Female (*n* = 416)	
	*N*	%	*N*	%	*n*	%
F2	2	0.28	1	0.33	1	0.24
F3	146	20.31	37	12.21	109	26.20
F4	313	43.53	126	41.58	187	44.95
F5	36	5.01	5	1.65	31	7.45
F6	5	0.70	0	0.00	5	1.20
F7	1	0.14	0	0.00	1	0.24
F8	1	0.14	1	0.33	0	0.00
F9	215	29.90	133	43.89	82	19.71
Internalising	558	77.61	190	62.71	368	88.46
Externalising	121	16.83	89	29.37	32	7.69
One Diagnosis	473	65.79	216	71.29	257	61.78
Multiple Diagnoses	246	34.21	87	28.71	159	38.22

F2 = Schizophrenia, Schizotypal and Delusional Disorders. F3 = Mood (Affective) Disorders. F4 - Neurotic, Stress-related and Somatoform Disorders. F5 = Behavioural Syndromes Associated with Physiological Disturbances and Physical Factors. F6 = Disorders of Adult Personality and Behaviour. F7 = Mental Retardation. F8 = Disorders of Psychological Development. F9 = Behavioural and Emotional Disorders with Onset Usually Occurring in Childhood and Adolescence. Internalising (F31, F32, F33, F34, F40, F41, F42, F43, F50, F51, F54, F93) and Externalising (F60, F63, F90, F91, F92) according to the hierarchical taxonomy of psychopathology (HiTOP) [34]

**Table 2:** Descriptives for emotional and behavioural difficulties (SDQ)

Scale	Report	Total	Gender	
			Male	Female
		*M* (*SD*)	*M* (*SD*)	*M* (*SD*)
Total Difficulties	Self	15.71 (5.90)	14.0 (5.77)	16.96 (5.69)
	Caregiver	15.60 (6.18)	16.42 (5.88)	14.97 (6.35)
Internalising Problems	Self	8.80 (4.11)	6.87 (3.64)	10.22 (3.84)
	Caregiver	8.46 (3.88)	7.85 (3.82)	8.95 (3.86)
Externalising Problems	Self	6.90 (3.43)	7.13 (3.43)	6.74 (3.42)
	Caregiver	7.14 (4.27)	8.57 (4.13)	6.02 (4.04)
Emotional Problems	Self	5.34 (2.88)	3.78 (2.53)	6.48 (2.57)
	Caregiver	5.43 (2.59)	4.75 (2.51)	5.96 (2.52)
Peer Problems	Self	3.46 (2.11)	3.09 (2.03)	3.73 (2.12)
	Caregiver	3.03 (2.22)	3.08 (2.26)	2.99 (2.19)
Hyperactivity/Inattention	Self	4.43 (2.27)	4.51 (2.24)	4.38 (2.29)
	Caregiver	4.14 (2.72)	5.10 (2.77)	3.38 (2.43)
Conduct Problems	Self	2.47 (1.74)	2.62 (1.74)	2.37 (1.73)
	Caregiver	3.00 (2.13)	3.47 (2.04)	2.64 (2.12)
Prosocial Behaviour	Self	7.80 (1.94)	7.43 (2.07)	8.07 (1.80)
	Caregiver	7.46 (1.91)	7.23 (1.88)	7.65 (1.91)

* M* Mean, *SD* Standard Deviation. Emotional and Behavioural Difficulties = Self- and Caregiver-Reports of the SDQ (The Strength and Difficulties Questionnaire)

### Prevalence of bullying and distressing bullying-specific intrusions

Table 3 shows frequencies of bullying experiences, regarding gender and types of bullying (direct, indirect, cyber). 29% of the outpatients were actively involved in bullying (22% victims, 5% bully-victims, 2% bullies). Cyberbullying was the least frequent type of bullying among victims (4%) and bullies (< 1%), respectively. Bullying frequency was partly gender-dependent: There were more female victims than male victims (ꭓ^2^ [1] = 4.71, *p* = .003, Cramers V = 0.081), more male direct bullies than female direct bullies (ꭓ^2^ [1] = 4.11, *p* = .043, Cramers V = 0.076) and more female indirect victims than male indirect victims (ꭓ^2^ [1] = 15.85, *p* < .001, Cramers V = 0.148). Out of all outpatients with experiences of bullying, 32% reported distressing bullying-specific intrusions. Female outpatients reported more distressing bullying-specific intrusions than male outpatients (ꭓ^2^ [1] = 7.44, *p* = .006, Cramers V = 0.102).

**Table 3:** Descriptives for peer bullying according to role of bullying, type of bulling and gender

Peer Bullying	Total (*N* = 719)		Gender				
			Male (*n* = 303)		Female (*n* = 416)		Gender Differences
	*n*	%	*n*	%	*n*	%	*p*-values
Bullies	17	2.36	10	3.30	7	1.68	0.245
Victims	155	21.56	53	17.49	102	24.52	0.030*
Bully-Victims	36	5.01	21	6.93	15	3.61	0.064
Direct Victims (item 1)	115	15.99	54	17.82	61	14.66	0.299
Direct Bullies (item 2)	41	5.70	24	7.92	17	4.09	0.043*
Indirect Victims (item 3)	130	18.08	34	11.22	96	23.08	< 0.001***
Indirect Bullies (item 4)	19	2.64	9	2.97	10	2.40	0.816
Cyber Victims (item 5)	31	4.31	14	4.62	17	4.09	0.871
Cyber Bullies (item 6)	6	0.83	5	1.65	1	0.24	0.102
Bullying Involvement (any of items 1-6)	208	28.93	84	27.72	124	29.81	0.599
Distressing Bullying-Specific Intrusions	72	10.01	19	6.27	53	12.74	0.006**

Peer Bullying = Items 1-6 from the Screening for Bullying Experiences. Gender differences examined by Chi²-Tests, *p*-values: <0.001***, <0.01**, <0.05*

### Differences in the likelihood of specific diagnoses (categorically assessed psychopathology)

**Fig. 2:**
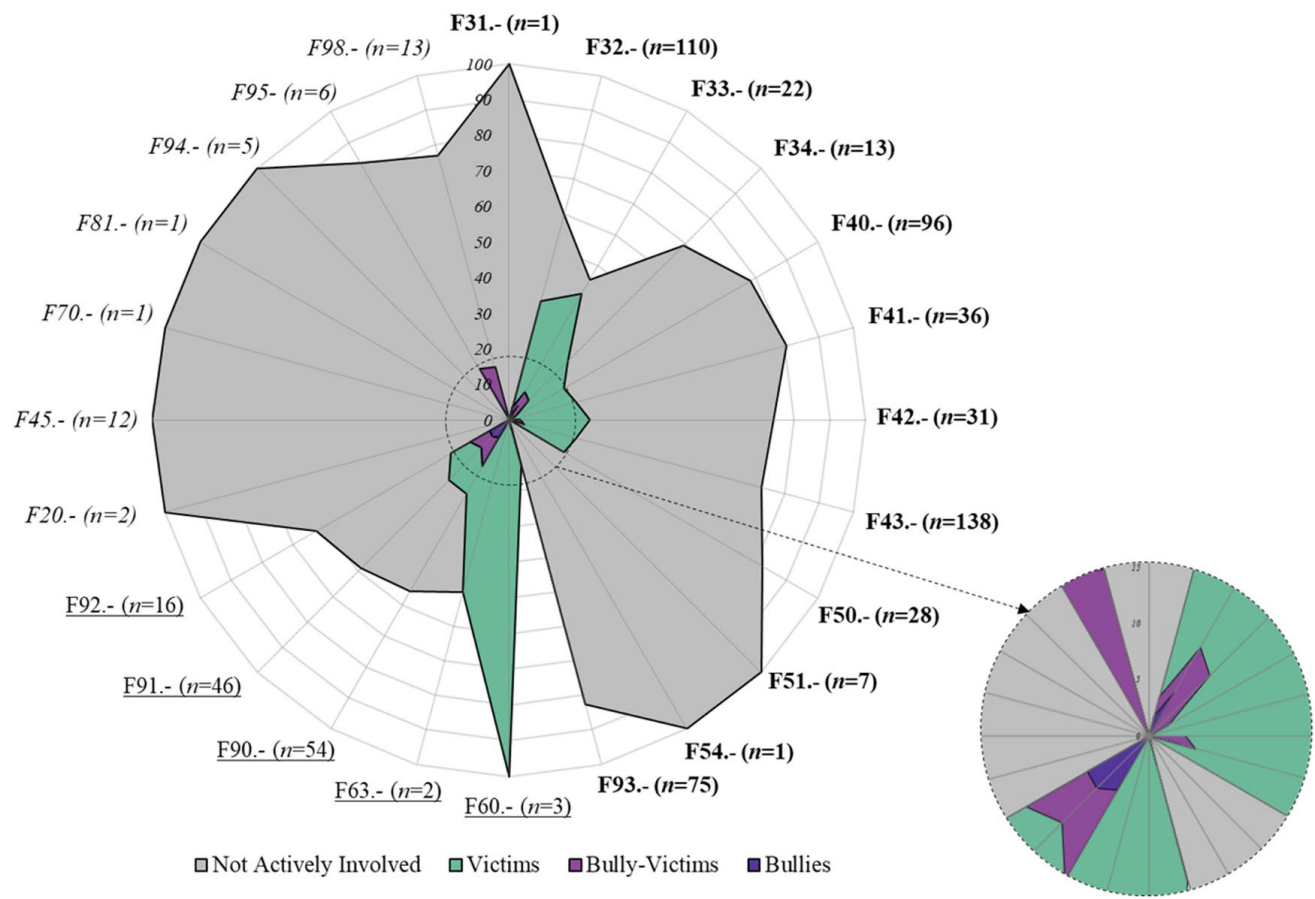
Radar Chart of the Relative Frequencies of Bullying Involvement in the ICD-10 Primary Diagnosis Subgroups Note. ICD-10 diagnoses after supervised structured clinical interviews (self- and caregiver-report) by trained mental health professionals. n = number of outpatients with each primary diagnosis subgroup. Primary diagnosis subgroups = categorised as internalising (F31, F32, F33, F34, F40, F41, F42, F43, F50, F51, F54, F93), externalising (F60, F63, F90, F91, F92), and other (F20, F45, F70, F81, F94, F95, F98) according to [34]

Table 4 presents frequencies of bullying roles (victim, bully-victim, bully) in the diagnosis groups. A descriptive radar chart presents the relative frequencies of bullying roles for all ICD-10 primary diagnosis subgroups (see Fig. 2). While some overlap was revealed, certain subgroups were highlighted for each bullying role (victims: F60, F63, F33, F32, bully-victims: F95, F98, F90, F92, bullies: F91, F92, F90, F33).

**Table 4:** Descriptives for ICD-10 primary diagnosis groups according to the outpatients’ bullying roles

	Bullies		Victims		Bully-Victims	
	Yes (*n* = 17)	No (*n* = 702)	Yes (*n* = 155)	No (*n* = 564)	Yes (*n* = 36)	No (*n* = 683)
	*n* (%)	*n* (%)	*n* (%)	*n* (%)	*n* (%)	*n* (%)
F2^4^	0 (0.00)	2 (0.28)	0 (0.00)	2 (0.35)	0 (0.00)	2 (0.29)
F3^3^	3 (17.65)	143 (20.37)	50 (32.26)	96 (17.02)	7 (19.44)	139 (20.35)
F4^3^	6 (35.29)	307 (43.73)	58 (37.42)	255 (45.21)	9 (25.00)	304 (44.51)
F5^4^	0 (0.00)	36 (5.13)	5 (3.23)	31 (5.50)	0 (0.00)	36 (5.27)
F6^4^	0 (0.00)	5 (0.71)	4 (2.58)	1 (0.18)	0 (0.00)	5 (0.73)
F7^4^	0 (0.00)	1 (0.14)	0 (0.00)	1 (0.18)	0 (0.00)	1 (0.15)
F8^4^	0 (0.00)	1 (0.14)	0 (0.00)	1 (0.18)	0 (0.00)	1 (0.15)
F9^2^	8 (47.06)	207 (29.49)	38 (24.52)	177 (31.38)	20 (55.56)	195 (28.55)
Internalising^1^	10 (58.82)	548 (78.06)	123 (79.35)	435 (77.13)	18 (50.00)	540 (79.06)
Externalising^2^	7 (41.18)	114 (16.24)	31 (20.00)	90 (15.96)	15 (41.67)	106 (15.52)
One Diagnosis^2^	7 (41.18)	466 (66.38)	98 (63.23)	375 (66.49)	25 (69.44)	448 (65.59)
Multiple Diagnoses^1^	10 (58.82)	236 (33.62)	57 (36.77)	189 (33.51)	11 (30.56)	235 (34.41)

Internalising (F31, F32, F33, F34, F40, F41, F42, F43, F50, F51, F54, F93) and Externalising (F60, F63, F90, F91, F92) according to the hierarchical taxonomy of psychopathology (HiTOP) [34]. Only predictors with *n* ≥ 10 in both categories (yes, no) were included in regression analyses, *p*-values: <0.001***, <0.01**, <0.05*. ^1^ With bully, victim, and bully-victim as predictors. ^2^ With victim and bully-victim as predictors. ^3^ With victim as predictor. ^4^ No logistic regression analysis possible

Binary logistic regression analyses were completed for cases with a minimum of *n* = 10 per bullying role x diagnosis combination [39]: victim as predictor for the outcomes F3 and F4; victim and bully-victim as predictors for the outcomes F9, externalising, and one diagnosis; victim, bully-victim, and bully as predictors for the outcomes internalising and multiple diagnoses (see footnotes in Table 4).

Victims had a higher likelihood of being diagnosed with a mood disorder (F3) than non-victims (b = 0.84, SE = 0.21, *p* < .001, OR = 2.32, 95% CI [1.55, 3.46]). However, victims were equally likely to receive the diagnosis of a neurotic, stress-related and somatoform disorder (F4; b = 0.32, SE = 0.19, *p* = .084, OR = 0.72, 95% CI [0.50, 1.04]) and the diagnosis of a behavioural and emotional disorder with onset in childhood and adolescence (F9; b = -0.26, SE = 0.21, *p* = .207, OR = 0.77, 95% CI [0.50, 1.15]). Being a victim was not associated with a higher risk of receiving an internalising diagnosis (b = -0.02, SE = 0.23, *p* = .937, OR = 0.98, 95% CI [0.64, 1.55]) or an externalising diagnosis (b = 0.41, SE = 0.24, *p* = .081, OR = 1.51, 95% CI [0.94, 2.38]), one diagnosis (b = -0.13, SE = 0.19, *p* = .481, OR = 0.87, 95% CI [0.60, 1.27]) or multiple diagnoses (b = 0.17, SE = 0.19, *p* = .369, OR = 1.19, 95% CI [0.81, 1.72]).

Bully-victims had a higher likelihood of being diagnosed with a F9 diagnosis than non-bully-victims (b = 1.08, SE = 0.35, *p* = .002, OR = 2.95, 95% CI [1.49, 5.93]). They also had an increased risk of externalising disorders compared to non-bully-victims (b = 1.46, SE = 0.36, *p* < .001, OR = 4.31, 95% CI [2.10, 8.70]) but a lower likelihood of internalising disorders (b = -1.36, SE = 0.35, *p* < .001, OR = 0.26, 95% CI [0.13, 0.51]). Being a bully-victim was not associated with a higher risk for receiving one diagnosis (b = 0.14, SE = 0.37, *p* = .698, OR = 1.16, 95% CI [0.57, 2.50]) or multiple diagnoses (b = -0.11, SE = 0.37, *p* = .774, OR = 0.90, 95% CI [0.42, 1.83]).

Bullies had a lower likelihood of being diagnosed with an internalising disorder than non-bullies (b = -1.01, SE = 0.50, *p* < .046, OR = 0.37, 95% CI [0.14, 1.03]) and a higher likelihood of being diagnosed with multiple diagnoses than non-bullies (b = 1.07, SE = 0.50, *p* = .033, OR = 2.92, 95% CI [1.10, 8.16]). Further tests regarding bullies could not be conducted (F2-F9, externalising disorders) due to low case numbers. The results of additional exploratory separate analyses for children (ages 6–9) and adolescents (ages 10–20) regarding differences in the likelihood of specific diagnoses are presented in the Supplementary Material M1.

### Differences in the extent of diagnosis-unspecific emotional and behavioural difficulties (dimensionally assessed psychopathology)

Table 5 presents descriptives of the SDQ scores for all bullying roles. Compared to non-victims, victims had higher self-reported total difficulties (F(1, 714) = 87.92, *p* < .001, partial η² = 0.110), internalising problems (F(1, 715) = 90.23, *p* < .001, partial η² = 0.112), externalising problems (F(1, 714) = 19.75, *p* < .001, partial η² = 0.027), emotional problems (F(1, 715) = 38.22, *p* < .001, partial η² = 0.051), peer problems (F(1, 716) = 96.36, *p* < .001, partial η² = 0.119), hyperactivity/inattention (F(1, 716) = 11.43, *p* = .001, partial η² = 0.016) and conduct problems (F(1, 714) = 18.46, *p* < .001, partial η² = 0.025), even after adjusting p-values (see Table 5). Similarly, they had also higher caregiver-reported total difficulties (F(1, 612) = 24.14, *p* < .001, partial η² = 0.038), internalising problems (F(1, 612) = 19.7, *p* < .001, partial η² = 0.031), externalising problems (F(1, 612) = 9.22, *p* = .003, partial η² = 0.015), emotional problems (F(1, 613) = 6.41, *p* = .012, partial η² = 0.010), peer problems (F(1, 613) = 23.08, *p* < .001, partial η² = 0.036), hyperactivity/inattention (F(1, 613) = 7.77, *p* = .005, partial η² = 0.013) and conduct problems (F(1, 613) = 6.24, *p* = .013, partial η² = 0.010), even after adjusting p-values (see Table 5).

Compared to non-bully-victims, bully-victims had higher self-reported total difficulties (F(1, 714) = 40.35, *p* < .001, partial η² = 0.053), internalising problems (F(1, 715) = 10.44, *p* = .001, partial η² = 0.014), externalising problems (F(1, 714) = 49.63, *p* < .001, partial η² = 0.065), peer problems (F(1, 716) = 22.29, *p* < .001, partial η² = 0.030), hyperactivity/inattention (F(1, 716) = 19.17, *p* < .001, partial η² = 0.026) and conduct problems (F(1, 714) = 65.88, *p* < .001, partial η² = 0.084). Further, they had lower self-reported prosocial behaviour (F(1, 716) = 10.05, *p* = .002, partial η² = 0.014). All effects remained significant even after p-values were adjusted (see Table 5). Accordingly, bully-victims had also higher caregiver-reported total difficulties (F(1, 612) = 21.60, *p* < .001, partial η² = 0.034), internalising problems (F(1, 612) = 6.013, *p* = .014, partial η² = 0.010), externalising problems (F(1, 612) = 19.99, *p* < .001, partial η² = 0.032), peer problems (F(1, 613) = 9.76, *p* = .002, partial η² = 0.016), hyperactivity/inattention (F(1, 613) = 14.04, *p* < .001, partial η² = 0.022) and conduct problems (F(1, 613) = 17.14, *p* < .001, partial η² = 0.027), even after adjusting p-values (see Table 5).

Compared to non-bullies, bullies showed higher self-reported externalising problems (F(1, 714) = 9.92, *p* = .002, partial η² = 0.014), hyperactivity/inattention (F(1, 716) = 5.51, *p* = .019, partial η² = 0.008) and conduct problems (F(1, 714) = 9.74, *p* = .002, partial η² = 0.013) and less self-reported prosocial behaviour (F(1, 716) = 8.99, *p* = .003, partial η² = 0.012). All found effects remained significant even after p-values were adjusted (see Table 5). Bullies also had higher caregiver-reported externalising problems (F(1, 612) = 4.60, *p* = .032, partial η² = 0.007) and conduct problems (F(1, 613) = 4.36, *p* = .037, partial η² = 0.007) but those differences were no longer statistically significant after adjusting p-values (adjusted *p*s = 0.149; see Table 5). The results of additional exploratory separate analyses for children (ages 6–9) and adolescents (ages 10–20) regarding differences in the extent of diagnosis-unspecific emotional and behavioural difficulties between those outpatients actively involved and those outpatients not actively involved in bullying are presented in the Supplementary Material M2.

**Table 5:** Descriptives for emotional and behavioural difficulties according to the outpatients’ bullying roles

Scale	Report	Bullying Roles								
		Bullies			Victims			Bully-Victims		
		Yes	No	Group differences	Yes	No	Group Diffe- Rences	Yes	No	Group differences
		*M* (*SD*)	*M* (*SD*)	*p*-value (adjusted)	*M* (*SD*)	*M* (*SD*)	*p*-value (adjusted)	*M* (*SD*)	*M* (*SD*)	*p*-value (adjusted)
Total Difficulties	Self	17.82 (4.19)	15.66 (5.93)	0.135 (0.217)	19.44 (5.97)	14.69 (5.46)	< 0.001*** (< 0.001***)	21.64 (6.12)	15.40 (5.73)	< 0.001*** (< 0.001***)
	Caregiver	17.33 (4.51)	15.56 (6.22)	0.273 (0.364)	17.94 (5.89)	14.98 (6.12)	< 0.001*** (< 0.001***)	20.47 (5.46)	15.34 (6.11)	< 0.001*** (< 0.001***)
Internalising Problems	Self	8.35 (3.48)	8.81 (4.12)	0.648 (0.740)	11.42 (4.02)	8.08 (3.83)	< 0.001*** (< 0.001***)	10.94 (3.86)	8.69 (4.09)	0.001** (0.002**)
	Caregiver	7.87 (3.80)	8.48 (3.88)	0.546 (0.546)	9.79 (3.97)	8.11 (3.78)	< 0.001*** (< 0.001***)	10.09 (3.73)	8.37 (3.87)	0.014* (0.019*)
Externalising Problems	Self	9.47 (2.18)	6.84 (3.43)	0.002** (0.008**)	7.97 (3.72)	6.61 (3.28)	< 0.001*** (< 0.001***)	10.69 (3.49)	6.70 (3.31)	< 0.001*** (< 0.001***)
	Caregiver	9.47 (2.97)	7.08 (4.28)	0.032* (0.149)	8.15 (4.01)	6.87 (4.30)	0.003** (0.005**)	10.38 (3.41)	6.96 (4.24)	< 0.001*** (< 0.001***)
Emotional Problems	Self	5.29 (2.71)	5.34 (2.88)	0.947 (0.947)	6.57 (2.72)	5.00 (2.83)	< 0.001*** (< 0.001***)	5.89 (2.61)	5.31 (2.89)	0.241 (0.241)
	Caregiver	4.47 (2.83)	5.45 (2.58)	0.145 (0.233)	5.94 (2.62)	5.29 (2.57)	0.012* (0.015*)	5.88 (2.41)	5.40 (2.60)	0.316 (0.352)
Peer Problems	Self	3.06 (1.56)	3.47 (2.12)	0.425 (0.566)	4.85 (2.19)	3.08 (1.92)	< 0.001*** (< 0.001***)	5.06 (2.23)	3.38 (2.07)	< 0.001*** (< 0.001***)
	Caregiver	3.40 (1.80)	3.02 (2.23)	0.516 (0.546)	3.85 (2.37)	2.81 (2.14)	< 0.001*** (< 0.001***)	4.22 (2.01)	2.97 (2.22)	0.002** (0.003**)
Hyperactivity/ Inattention	Self	5.71 (1.83)	4.40 (2.27)	0.019* (0.038*)	4.97 (2.43)	4.28 (2.21)	0.001** (0.001**)	6.03 (2.13)	4.35 (2.25)	< 0.001*** (< 0.001***)
	Caregiver	5.33 (2.47)	4.11 (2.72)	0.085 (0.204)	4.73 (2.69)	3.98 (2.71)	0.005** (0.009**)	5.88 (2.23)	4.04 (2.72)	< 0.001*** (< 0.001***)
Conduct Problems	Self	3.76 (1.09)	2.44 (1.74)	0.002** (0.008**)	3.00 (1.93)	2.33 (1.65)	< 0.001*** (< 0.001***)	4.67 (1.96)	2.36 (1.65)	< 0.001*** (< 0.001***)
	Caregiver	4.13 (1.60)	2.98 (2.13)	0.037* (0.149)	3.42 (2.08)	2.90 (2.13)	0.013 * (0.015*)	4.50 (1.93)	2.92 (2.11)	< 0.001*** (< 0.001***)
Prosocial Behaviour	Self	6.41 (1.80)	7.83 (1.93)	0.003** (0.008**)	7.86 (1.97)	7.78 (1.94)	0.672 (0.672)	6.81 (2.19)	7.85 (1.92)	0.002** (0.002**)
	Caregiver	6.67 (1.54)	7.48 (1.91)	0.102 (0.204)	7.36 (1.96)	7.49 (1.89)	0.514 (0.514)	7.16 (1.72)	7.48 (1.92)	0.352 (0.352)

*M* Mean. *SD* Standard Deviation. Emotional and Behavioural Difficulties = Self- and Caregiver-Reports of the SDQ (The Strength and Difficulties Questionnaire). Group differences examined by ANOVA, *p*-values: <0.001***, <0.01**, <0.05*. Adjusted = Adjusted *p*-values using the Benjamini-Hochberg procedure to control False Discovery Rate

## Discussion

This study provides evidence for the association between bullying and both diagnosis-specific and transdiagnostic patterns of psychopathology in a large sample of child and adolescent psychotherapy outpatients. The prevalence (Question 1) of 22% victims and 5% bully-victims in our clinical sample was descriptively higher than that of school bullying (9% victims, 3% bullies, 2% bully-victims) found in the general population of German pupils [6]. The identified 2% bullies aligns with the comparatively little perpetration found in other clinical samples [19, 20]. Overall, 29% of outpatients reported active bullying involvement in the last six months. While not empirically tested, the seemingly rather low prevalence of cyberbullying highlights the importance of addressing traditional forms of bullying, especially in clinical samples [19, 40].

The exploratory finding that 32% of those actively involved in bullying during the last six months reported distressing bullying-specific intrusions, highlights some of the possible bullying-related psychological burden in clinical populations. This supports the proposal of peer victimisation as a potentially traumatic event [41] and the finding that bullying victimisation was rated as the most emotional distressing adverse childhood experience out of at least two [42]. As bullying-related psychological burden is not regularly addressed in prevention nor is it routinely assessed in psychotherapeutic settings, current processes and tools may need advancement.

Regarding the categorically assessed psychopathology (Question 2a), victims had more than twice the odds of being diagnosed with a mood disorder (F3) compared to non-victims, partly supporting our hypothesis and consistent with [18]. However, victims were not more likely to overall be diagnosed with internalising or externalising disorders, contradicting our hypothesis. This may reflect differences in diagnosis composition: mood disorders accounted for 26% of primary internalising diagnoses here, whereas previous research did not distinguish between specific internalising diagnoses or between primary and secondary diagnoses [28]. Additional clarification may lie in sample composition: previous research reported externalising disorders as most prevalent, whereas over three-quarters of our sample had internalising primary diagnoses [21]. Thus, findings on psychopathology and bullying may not be easily generalisable, even within clinical samples. Bully-victims were four times more likely than non-bully-victims to receive an externalising diagnosis, aligning with our hypothesis and previous findings, and almost 3 times more likely to receive an F9 diagnosis (i.e., comprising internalising and externalising aspects), supporting previous research [21, 28]. Their lower likelihood of internalising disorders was not reported in previous clinical studies. Finally, bullies were less likely than non-bullies to receive an internalising disorder, contrary to our hypothesis and previous clinical samples [18, 21, 28]. However, they had nearly three times the odds of multiple diagnoses, a novel finding consistent with reports of high psychological burden in bullies [18, 27].

Regarding the dimensionally assessed psychopathology (Question 2b), elevated internalising and emotional problems scores for victims compared to non-victims confirm our hypothesis and corroborate previous findings [28]. Additional elevated scores confirm the heterogenous patterns found in previous clinical samples [18, 21, 28]. Furthermore, the bully-victims’ greater emotional and behavioural difficulties and lower prosocial behaviour align with our hypothesis and support previous findings in clinical samples [21, 28]. The highest scores for total difficulties in bully-victims correspond to previously reported findings [21]. Finally, the greater self-reported emotional and behavioural difficulties and lower prosocial behaviour in bullies, compared to non-bullies, support our hypothesis and previous clinical samples [18, 21, 28]. Interestingly, self- and caregiver-reported emotional and behavioural difficulties were similar for victims and bully-victims but differed decidedly for bullies. This is especially surprising as the high levels of externalising problems self-reported by the bullies are usually more likely to be perceived by caregivers. Several explanations are possible, e.g., bullies in clinical populations may experience their behaviour as more problematic or less controllable than caregivers recognise. Future studies could clarify the somewhat hazy picture of bullying perpetration and psychopathology.

To summarise, the findings went beyond linking bullying involvement to specific categorial diagnoses (Question 2): While victims were more likely to have a mood disorder, they showed more internalising and more externalising diagnosis-unspecific emotional and behavioural difficulties than non-victims. Bully-victims were less likely to have internalising disorders but more likely to have externalising disorders or a F9 diagnosis than non-bully-victims which corresponds to their reported internalising and externalising diagnosis-unspecific emotional and behavioural difficulties. Bullies were less likely to have an internalising disorder and more likely to have multiple diagnoses than non-bullies which does not clearly correspond to more self-reported externalising diagnosis-unspecific emotional and behavioural difficulties. This suggests the link between bullying and psychopathology to be only partly attributable to specific disorders, pointing to heterogeneous and complex symptom patterns. Addressing bullying in clinical care may therefore benefit from focusing on transdiagnostic bullying-related psychological burden alongside specific diagnoses.

Limitations include the cross-sectional design and the lack of a comparison group, which preclude causal or directional interpretations. The findings are not generalisable to the general population or school populations. Comparisons with other previously published samples are descriptive in nature and should be interpreted cautiously, as differences in recruitment, sample composition, and assessment procedures limit direct comparability. Due to potential referral and selection biases inherent to clinical populations and the setting, the findings may not be representative and are limited to CBT treatment-seeking outpatient populations. Despite the relatively large sample for a clinical context, the number of outpatients was insufficient for some intended sub-analyses. While required for certain analyses, grouping diagnoses inevitably entails some loss of information and blurs diagnostic heterogeneity. Although poor mental health outcomes may occur after bullying has stopped [43], only current bullying was examined. As patients were asked about distressing bullying-specific intrusions only if they reported involvement as a victim, bully-victim, or bully in the past six months, the bullying-related psychological burden reported here may be underestimated (e.g., earlier experiences or witnessing bullying). Intrusions are a well-recognised feature of trauma-related burden, but they represent only one manifestation of psychological burden. Other bullying-related presentations should be explored in future studies and assessed in clinical settings.

From a practical perspective, bullying and its related psychological burden should be routinely assessed in mental health care settings. Assessments should include current and lifetime victimisation and perpetration, extend beyond specific disorders, and address manifestations of self-reported bullying-related psychological burden. To integrate this into treatment planning, clinicians’ awareness must be strengthened. Addressing bullying-related mental health outcomes in bullying prevention and intervention may also facilitate more timely support. Future research should differentiate between school and clinical samples and adopt longitudinal, disorder-specific and transdiagnostic approaches. Developing and testing bullying-informed therapeutic interventions should be prioritised, incorporating the perspectives of affected children and adolescents as experts.

## Conclusions

The present study provides a systematic investigation of current involvement as a victim, bully-victim or bully and categorially and dimensionally assessed psychopathology in child and adolescent psychotherapy outpatients. It includes a large clinical sample, covers a broad range of psychological disorders and applies gold-standard diagnostics as well as a multi-informant approach. The findings underscore the clinical relevance of bullying involvement with partly diagnosis-specific and partly transdiagnostic associations and the importance of addressing bullying in child and adolescent psychotherapy.

Outpatients currently involved as victims, bully-victims, and bullies had higher likelihoods of certain specific diagnoses and reported greater diagnosis-unspecific emotional and behavioural difficulties than non-involved outpatients. There is evidence for distressing bullying-specific intrusions and bullying role-specific psychopathology, supporting the practice-informed need to routinely screen for bullying and related psychological burden in mental health care settings.

Future research could further clarify diagnosis-specific and -unspecific mechanisms between bullying and psychopathology and develop bullying-specific effective psychotherapeutic approaches. Integrating bullying-informed assessment and interventions into routine clinical practice may potentially facilitate the identification of needs, inform treatment planning, and strengthen prevention efforts.

## Supplementary Information

Below is the link to the electronic supplementary material.

Supplementary Material 1.

## Data Availability

The dataset analysed during the current study is not publicly available due to ethical and consent restrictions related to the clinical sample but may be available from the corresponding author on reasonable request.

## Abbreviations

FBZ: Mental Health Research and Treatment Centre
DZPG: German Centre for Mental Health
CBT: Cognitive behavioural therapy
Kinder-DIPS: Diagnostic interview for mental disorders in children and adolescents
HiTOP: The hierarchical taxonomy of psychopathology
SDQ: The strength and difficulties questionnaire
F3: Mood disorder
F4: Neurotic, stress-related and somatoform disorder
F9: Behavioural and emotional disorder with onset in childhood and adolescence
