# Supplementary material for: Not all wounds are visible: Peer bullying and psychopathology in child and adolescent psychotherapy outpatients
Source: Child Adolesc Psychiatry Ment Health. 2026 Jul 29;20:101. doi: 10.1186/s13034-026-01146-w (PMC13452099; doi:10.1186/s13034-026-01146-w)
Supplement: Supplementary file 1 [file 13034_2026_1146_MOESM1_ESM.pdf]

Supplementary Material

1. Supplementary Material M1: Separate Analyses for Children and Adolescents Regarding Differences in the Likelihood of Specific Diagnoses (Categorically Assessed Psychopathology)
2. Supplementary Material M2: Separate Analyses for Children and Adolescents Regarding Differences in the Extent of Diagnosis-Unspecific Emotional and Behavioural Difficulties (Dimensionally Assessed Psychopathology)
3. References

Supplementary Material M1

Separate Analyses for Children and Adolescents Regarding Differences in the Likelihood of Specific Diagnoses (Categorically Assessed Psychopathology)

In line with the main analyses reported in the manuscript, each bullying role (victim, bully-victim, bully vs. not experienced as the reference category) was exploratorily tested as predictor of each diagnosis group (binary logistic regression analyses) within the subgroups of children (ages 6 – 9; n = 114) and adolescents (ages 10 – 20; n = 605) separately. Supplementary Tables T1 and T2 present frequencies of bullying in the diagnosis groups according to being a child or an adolescent. As with the main analyses reported in the manuscript, outcomes included F2-F9 as primary diagnosis, internalising primary diagnosis, externalising primary diagnosis, one diagnosis, as well as multiple diagnoses. Analyses were completed for cases with a minimum of $n = 10$ per bullying role x diagnosis combination [1]. This allowed for the exploration of victim as predictor for the outcomes F9, internalising, externalising, and one diagnosis within the children and for exploring victim as predictor for the outcomes F3, F4, externalising, and multiple diagnoses as well as victim and bully-victim as predictors for the outcomes F9, internalising, and one diagnosis within the adolescents (see footnotes in Supplementary Tables T1 and T2). No further analyses could be conducted due to low case numbers.

Child victims had a higher likelihood of being diagnosed with an externalising disorder than child non-victims (b = 1.02, SE = 0.46, $p = .025$, OR = 2.78, 95% CI [1.14, 6.93]). However, being a child victim was not associated with a higher risk of receiving an F9 diagnosis (b = 0.08, SE = 0.47, $p = .870$, OR = 1.08, 95% CI [0.44, 2.79]), an internalising diagnosis (b = -0.58, SE = 0.45, $p = .195$, OR = 0.56, 95% CI [0.23, 1.34]) or one single diagnosis (b = 0.07, SE = 0.50, p = .882, OR = 1.08, 95% CI [0.42, 3.04]).

Adolescent victims had a higher likelihood of having an F3 diagnosis than adolescent non-victims (b = 0.93, SE = 0.22, $p < .001$, OR = 2.52, 95% CI [1.65, 3.84]). Being an adolescent victim was not associated with a higher risk of being diagnosed with an F4 diagnosis (b = -0.39, SE = 0.20, $p = .054$, OR = 0.68, 95% CI [0.45, 1.00]), an F9 diagnosis (b = -0.51, SE = 0.26, $p = .054$, OR = 0.60, 95% CI [0.35, 0.99]), an internalising disorder (b = 0.38, SE = 0.29, $p = .185$, OR = 1.46, 95% CI [0.85, 2.65]), an externalising disorder (b = -0.02, SE = 0.29, $p = .937$, OR = 0.98, 95% CI [0.54, 1.70]), one single diagnosis (b = -0.16, SE = 0.21, $p = .437$, OR = 0.85, 95% CI [0.57, 1.28]) or multiple diagnoses (b = 0.19, SE = 0.20, $p = .365$, OR = 1.20, 95% CI [0.80, 1.79]) than adolescent non-victims.

Adolescent bully-victims had a higher likelihood of being diagnosed with an F9 diagnosis (b = 0.89, SE = 0.42, p = .033, OR = 2.44, 95% CI [1.05, 5.51]) and a lower likelihood of having an internalising disorder (b = -0.93, SE = 0.43, p = .032, OR = 0.40, 95% CI [0.17, 0.96]) than adolescent non-bully-victims. Being an adolescent bully-victim was not associated with a higher risk of being diagnosed with one single diagnosis (b = 0.54, SE = 0.48, p = .264, OR = 1.71, 95% CI [0.71, 4.77]) than adolescent non-bully-victims.

Supplementary Table T1

Descriptives for ICD-10 Primary Diagnosis Groups According to the Childrens' Bullying Roles

	Bullies		Victims		Bully-Victims	
	Yes	No	Yes	No	Yes	No
	(*n* = 5)	(*n* = 109)	(*n* = 26)	(*n* = 88)	(*n* = 11)	(*n* = 103)
	n (%)	*n* (%)	*n* (%)	*n* (%)	*n* (%)	*n* (%)
F2[2]	0 (0.00)	0 (0.00)	0 (0.00)	0 (0.00)	0 (0.00)	0 (0.00)
F3[2]	0 (0.00)	4 (3.67)	1 (3.85)	3 (3.41)	0 (00.00)	4 (3.88)
F4[2]	2 (40.00)	31 (28.44)	8 (30.77)	25 (28.41)	2 (18.18)	31 (30.10)
F5[2]	0 (0.00)	4 (3.67)	0 (0.00)	4 (4.55)	0 (0.00)	4 (3.88)
F6[2]	0 (0.00)	0 (0.00)	0 (0.00)	0 (0.00)	0 (0.00)	0 (0.00)
F7[2]	0 (0.00)	0 (0.00)	0 (0.00)	0 (0.00)	0 (0.00)	0 (0.00)
F8[2]	0 (0.00)	0 (0.00)	0 (0.00)	0 (0.00)	0 (0.00)	0 (0.00)
F9[1]	3 (60.00)	70 (64.22)	17 (65.38)	56 (63.64)	9 (81.82)	64 (62.14)
Internalising[1]	2 (40.00)	59 (54.13)	11 (42.31)	50 (56.82)	2 (18.18)	59 (57.28)
Externalising[1]	3 (60.00)	37 (33.94)	14 (53.85)	26 (29.55)	7 (63.64)	33 (32.04)
One Diagnosis[1]	3 (60.00)	79 (72.48)	19 (73.08)	63 (71.59)	6 (54.55)	76 (73.79)
Multiple Diagnoses[2]	2 (40.00)	30 (27.52)	7 (26.92)	25 (28.41)	5 (45.45)	27 (26.21)

Note. Internalising (F31, F32, F33, F34, F40, F41, F42, F43, F50, F51, F54, F93) and Externalising (F60, F63, F90, F91, F92) according to the hierarchical taxonomy of psychopathology (HiTOP) [34]. Only predictors with n ≥ 10 in both categories (yes, no) were included in regression analyses, *p*-values: <.001***, <.01**, <.05*. [1] With victim as predictor. [2] No logistic regression analysis possible.

Supplementary Table T2

Descriptives for ICD-10 Primary Diagnosis Groups According to the Adolescents' Bullying Roles

	Bullies		Victims		Bully-Victims	
	Yes (n = 12)	No (n = 593)	Yes (n = 129)	No (n = 476)	Yes (n = 25)	No (n = 580)
	n (%)	n (%)	n (%)	n (%)	n (%)	n (%)
F2[3]	0 (0.00)	2 (0.34)	0 (0.00)	2 (0.42)	0 (0.00)	2 (0.34)
F3[1]	3 (25.00)	139 (23.44)	49 (37.98)	93 (19.54)	7 (28.00)	135 (23.28)
F4[1]	4 (33.33)	276 (46.54)	50 (38.76)	230 (48.32)	7 (28.00)	273 (47.07)
F5[3]	0 (0.00)	32 (5.40)	5 (3.88)	27 (5.67)	0 (0.00)	32 (5.52)
F6[3]	0 (0.00)	5 (0.84)	4 (3.10)	1 (0.21)	0 (0.00)	5 (0.86)
F7[3]	0 (0.00)	1 (0.17)	0 (0.00)	1 (0.21)	0 (0.00)	1 (0.17)
F8[3]	0 (0.00)	1 (0.17)	0 (0.00)	1 (0.21)	0 (0.00)	1 (0.17)
F9[2]	5 (41.67)	137 (23.10)	21 (16.28)	121 (25.42)	11 (44.00)	131 (22.59)
Internalising[2]	8 (66.67)	489 (82.46)	112 (86.82)	385 (80.88)	16 (64.00)	481 (82.93)
Externalising[1]	4 (33.33)	77 (12.98)	17 (13.18)	64 (13.45)	8 (32.00)	73 (12.59)
One Diagnosis[1]	4 (33.33)	387 (65.26)	79 (61.24)	312 (65.55)	19 (76.00)	372 (64.14)
Multiple Diagnoses[1]	8 (66.67)	206 (34.74)	50 (38.76)	164 (34.45)	6 (24.00)	208 (35.86)

Note. Internalising (F31, F32, F33, F34, F40, F41, F42, F43, F50, F51, F54, F93) and Externalising (F60, F63, F90, F91, F92) according to the hierarchical taxonomy of psychopathology (HiTOP) [34]. Only predictors with n ≥ 10 in both categories (yes, no) were included in regression analyses, p-values: <.001***, <.01**, <.05*. [1] With victim as predictor. [2] With victim and bully-victim as predictors. [3] No logistic regression analysis possible.

Supplementary Material 2

Separate Analyses for Children and Adolescents Regarding Differences in the Extent of Diagnosis-Unspecific Emotional and Behavioural Difficulties (Dimensionally Assessed Psychopathology)

In line with the main analyses reported in the manuscript, group differences regarding the Strength and Difficulties Questionnaire (SDQ) scores between outpatients actively involved in bullying as a victim, bully-victim, or bully and those not actively involved were exploratorily examined (multiple one-way ANOVAs) within the groups of children (ages 6 – 9; n = 114) and adolescents (ages 10 – 20; n = 605) separately. As in the manuscript, outcomes included total difficulties, internalising problems, externalising problems, as well as all SDQ subscale scores and p-values were adjusted using the Benjamini-Hochberg procedure to control for the increased risk of Type I error due to multiple testing [2].

Child victims had higher self-reported total difficulties (F(1, 111) = 10.48, p = .002, adjusted p = .007, partial η^2 = .086), internalising problems (F(1, 112) = 9.56, p = .003, adjusted p = .007, partial η^2 = .079), externalising problems (F(1, 111) = 4.81, p = .030, adjusted p = .049, partial η^2 = .042), peer problems (F(1, 112) = 9.77, p = .002, adjusted p = .007, partial η^2 = .080), and conduct problems (F(1, 111) = 6.38, p = .013, adjusted p = .026, partial η^2 = .054) than child non-victims. Those effects all remained significant even after p-values were adjusted. Child victims also had higher self-reported emotional problems (F(1, 112) = 4.21, p = .043, adjusted p = .057, partial η^2 = .036) than child non-victims, but those differences were no longer statistically significant after adjusting p-values. Additionally, child victims had higher caregiver-reported total difficulties (F(1, 109) = 4.68, p = .033, adjusted p = .087, partial η^2 = .041), externalising problems (F(1, 109) = 4.86, p = .030, adjusted p = .087, partial η^2 = .043), and conduct problems (F(1, 110) = 5.21, p = .024, adjusted p = .087, partial η^2 = .045) than child non-victims, but those differences were no longer statistically significant after p-values were adjusted.

Compared to child non-bully-victims, child bully-victims had higher self-reported total difficulties ($F(1, 111) = 11.90$, $p = .001$, adjusted $p = .005$, partial $\eta^2 = .097$), internalising problems ($F(1, 112) = 11.12$, $p = .001$, adjusted $p = .005$, partial $\eta^2 = .090$), externalising problems ($F(1, 111) = 6.38$, $p = .013$, adjusted $p = .017$, partial $\eta^2 = .054$), emotional problems ($F(1, 112) = 6.73$, $p = .011$, adjusted $p = .017$, partial $\eta^2 = .057$), peer problems ($F(1, 112) = 7.99$, $p = .006$, adjusted $p = .011$, partial $\eta^2 = .067$), and conduct problems ($F(1, 111) = 9.40$, $p = .003$, adjusted $p = .007$, partial $\eta^2 = .078$). Child bully-victims also self-reported lower prosocial behaviour ($F(1, 112) = 5.06$, $p = .026$, adjusted $p = .030$, partial $\eta^2 = .043$) than child non-bully-victims. All the above effects remained significant even after p-values were adjusted. Furthermore, child bully-victims had higher caregiver-reported total difficulties ($F(1, 109) = 4.28$, $p = .041$, adjusted $p = .163$, partial $\eta^2 = .038$) and peer problems ($F(1, 109) = 5.72$, $p = .019$, adjusted $p = .148$, partial $\eta^2 = .050$) than child non-bully-victims, but these differences were no longer statistically significant after adjusting p-values.

No significant differences between child bullies and child non-bullies were found regarding any of the self-reported SDQ scores. Child bullies had lower caregiver-reported emotional problems ($F(1, 110) = 4.94$, $p = .028$, adjusted $p = .227$, partial $\eta^2 = .043$) than child non-bullies, but this effect was no longer statistically significant after p-values were adjusted.

Adolescent victims had higher self-reported total difficulties ($F(1, 601) = 79.25$, $p < .001$, adjusted $p < .001$, partial $\eta^2 = .117$), internalising problems ($F(1, 601) = 88.29$, $p < .001$, adjusted $p < .001$, partial $\eta^2 = .128$), externalising problems ($F(1, 601) = 15.11$, $p < .001$, adjusted $p < .001$, partial $\eta^2 = .025$), emotional problems ($F(1, 601) = 37.77$, $p < .001$, adjusted $p < .001$, partial $\eta^2 = .059$), peer problems ($F(1, 602) = 89.44$, $p < .001$, adjusted $p < .001$, partial $\eta^2 = .129$), hyperactivity/inattention ($F(1, 602) = 9.37$, $p = .002$, adjusted $p = .003$, partial $\eta^2 = .015$), and conduct problems ($F(1, 601) = 12.83$, $p < .001$, adjusted $p < .001$, partial $\eta^2 = .021$) than adolescent non-victims. All of those effects remained significant after p-values were adjusted. Similarly, adolescent victims had higher caregiver-reported total

difficulties (F(1, 501) = 19.07, $p < .001$, adjusted $p < .001$, partial $\eta^2 = .037$), internalising problems (F(1, 501) = 20.62, $p < .001$, adjusted $p < .001$, partial $\eta^2 = .040$), externalising problems (F(1, 501) = 4.98, $p = .026$, adjusted $p = .036$, partial $\eta^2 = .010$), emotional problems (F(1, 501) = 8.35, $p = .004$, adjusted $p = .008$, partial $\eta^2 = .016$), peer problems (F(1, 502) = 20.99, $p < .001$, adjusted $p < .001$, partial $\eta^2 = .040$), and hyperactivity/inattention (F(1, 502) = 4.94, $p = .027$, adjusted $p = .036$, partial $\eta^2 = .010$) than adolescent non-victims. All the above effects remained significant after p-values were adjusted.

Adolescent bully-victims had higher self-reported total difficulties (F(1, 601) = 30.60, $p < .001$, adjusted $p < .001$, partial $\eta^2 = .048$), internalising problems (F(1, 601) = 6.37, $p = .012$, adjusted $p = .016$, partial $\eta^2 = .010$), externalising problems (F(1, 601) = 41.44, $p < .001$, adjusted $p < .001$, partial $\eta^2 = .064$), peer problems (F(1, 602) = 17.30, $p < .001$, adjusted $p < .001$, partial $\eta^2 = .028$), hyperactivity/inattention (F(1, 602) = 16.72, $p < .001$, adjusted $p < .001$, partial $\eta^2 = .027$), and conduct problems (F(1, 601) = 53.05, $p < .001$, adjusted $p < .001$, partial $\eta^2 = .081$) than adolescent non-bully-victims. Adolescent bully-victims also self-reported lower prosocial behaviour (F(1, 602) = 4.80, $p = .029$, adjusted $p = .033$, partial $\eta^2 = .008$) than adolescent non-bully-victims. All of these effects remained significant after p-values were adjusted. In addition, adolescent bully-victims had higher caregiver-reported total difficulties (F(1, 501) = 16.29, $p < .001$, adjusted $p = .001$, partial $\eta^2 = .031$), internalising problems (F(1, 501) = 5.51, $p = .019$, adjusted $p = .026$, partial $\eta^2 = .011$), externalising problems (F(1, 501) = 14.37, $p < .001$, adjusted $p = .001$, partial $\eta^2 = .028$), peer problems (F(1, 502) = 6.11, $p = .014$, adjusted $p = .022$, partial $\eta^2 = .012$), hyperactivity/inattention (F(1, 502) = 10.23, $p = .001$, adjusted $p = .003$, partial $\eta^2 = .020$), and conduct problems (F(1, 501) = 12.20, $p = .001$, adjusted $p = .001$, partial $\eta^2 = .024$) than adolescent non-bully-victims. Those effects all remained significant after adjusting p-values.

Adolescent bullies had higher self-reported externalising problems (F(1, 601) = 6.93, $p = .009$, adjusted $p = .023$, partial $\eta^2 = .011$) and conduct problems (F(1, 601) = 7.35, $p = .007$,

adjusted p = .023, partial η^2 = .012) and lower self-reported prosocial behaviour ($F(1, 602)$ = 11.32, p = .001, adjusted p = .007, partial η^2 = .018) than adolescent non-bullies. These effects remained significant after p-values were adjusted. Furthermore, adolescent bullies had higher caregiver-reported externalising problems ($F(1, 501)$ = 5.28, p = .022, adjusted p = .071, partial η^2 = .010) and lower self-reported prosocial behaviour ($F(1, 502)$ = 4.95, p = .027, adjusted p = .071, partial η^2 = .010) than adolescent non-bullies, but those effects were no longer statistically significant after p-values were adjusted. Adolescent bullies also had higher caregiver-reported conduct problems ($F(1, 501)$ = 7.75, p = .006, adjusted p = .045, partial η^2 = .015). This effect remained significant even after adjusted p-values.

References

1. Vittinghoff E, McCulloch CE. Relaxing the rule of ten events per variable in Logistic and Cox Regression. *Am J Epidemiol*. 2007;165(6):710–718. doi:10.1093/aje/kwk052.
2. Benjamini Y, Hochberg Y. Controlling the False Discovery Rate: A practical and powerful approach to multiple testing. *J R Stat Soc Series B Stat Methodol*. 1995;57(1):289–300. doi:10.1111/j.2517-6161.1995.tb02031.x.
